# Interesting facts regarding the marginal mandibular branch of the facial nerve

**DOI:** 10.4103/0970-0358.73477

**Published:** 2010

**Authors:** Farida Hussan, Srijit Das

**Affiliations:** Department of Anatomy, Universiti Kebangsaan Malaysia, Jalan Raja Muda Abd Azia, 50300 Kuala Lumpur, Malaysia

Sir,

We read with much interest the article titled ’Marginal branch of the facial nerve: An anatomical study’ by Batra *et al*.[1] The cadaveric study describes the topographical anatomy of the marginal mandibular branch (MMB) of the facial nerve. We too would like to share some important facts related to the nerve branch.

According to standard textbook of anatomy, the MMB bears an important relationship with the lower border of the mandible.[2] This fact has been described by the authors. An important aspect is the effective and correct morphometric measurement of the anatomical structures. Perhaps the exact tools used for measurement were not highlighted properly by the authors. There is a shrinkage factor in all tissues after death and many measurements may not be the same compared to that in the living state.

The authors describe the facial artery to be an important landmark. It may be mentioned that while exploring a facial artery which is a deeper structure, a superficial nerve like MMB may already have been cut. A very important structure which is more superficial and has an important relationship with the MMB is the retromandibular vein. Interestingly, studies have described the retromandibular vein and the facial nerve to lie in the same fascial plane and prior knowledge of this fact may be beneficial for surgeons.[3]

The results of the study show that in all cases (100%), the MMB passed superficial to the facial artery which may be a debatable fact. Could the results be related to the particular ethnic group in India or the Indian population in general? Research reports even depict that MMB may also pass deep to the facial artery.[3] In case, there is variation regarding the MMB being either superficial or deep, the surgeon may have to be extra cautious.

It is pertinent to mention that the relationship of MMB with the inferior border of the mandible may be influenced by the sloping angle of the inferior border of the mandible. Interestingly, a danger zone for incision has been reported to be different in males and females.[4] Even age changes can alter the shape of mandible. The mandible dimensions may be different for each sex, i.e. male or female. The present article did not take into account the age and sex of the cadavers which can influence the results.

Overall, it is an interesting and enjoyable article with important clinical implications. We highly appreciate the sincere work of the authors and the Editor for publishing such informative articles which may be beneficial for surgeons.
